# Our Chicago Letter

**Published:** 1885-07

**Authors:** J. W. McLaughlin

**Affiliations:** Texas Member of Committee; Chicago, Ills.


### CORRESPONDENCE.

### The Chicago Meeting.

*Letter from Dr. J. W. McLaughlin, Texas Member of Committee.*

CHICAGO, ILLS., June 26, 1885.

DR. F. E. DANIEL,

TEXAS MEDICAL JOURNAL.

*Dear Doctor:*—The Committee on the International Medical Congress met in the parlors of the Palmer House, in this city, on the 24th inst., and adjourned today.

High legal authority having given the opinion that the action of the A. M. A. is legal, and that the committee, as now constituted, absorbs the original committee, all parties are satisfied, and there is quite a full attendance—about thirty members being present, including Drs. Billings and Hays, of the original seven. Dr. Flint resigned his place on the committee, and Dr. Gouley was appointed in his stead. An election for permanent officers of the committee resulted in the choice of Dr. R. Beverly Cole, of San Francisco, Cal., president, or chairman, and Dr. John V. Shoemaker, secretary. Those had been the temporary officers. A vice-chairman was also elected, Dr. Lynch, of Maryland, and it was resolved that in future, fifteen shall constitute a quorum. The work of revision of the list of appointments on the sections of the International Medical Congress was delegated to a sub-committee of nine, as follows: Scott, of Ohio; Wathen, of Ky.; Billings, U. S. A.; Shoemaker, of Pa.; Hamilton, M. H. S.; Linthicum, of Ark.; Upham, of Vt.; Gouley, of N. Y., and McLaughlin, of Texas.

I send you herewith a partial list of appointees. You will observe, Doctor, that not a member of this committee was placed in any position, for obvious reasons, (with one exception—Dr. Battey, of Ga., of worldwide renown). You will also notice that there has been a fair redistribution of appointments covering pretty thoroughly all States and Territories represented in the A. M. A., and wherever the name of a new code man occurred on the list, it was promptly erased, and the name of one known to be in affiliation with the association, and a supporter of the code of ethics inserted.

Texas is fairly represented. The appointees from that State being Dr. F. E. Daniel, Secretary on Section of Dermatology and Syphilis. Dr. Hardaway, of St. Louis, is the chairman of the section. You will have big work to do. Dr. Cupples is on the Section of Medical Education, Legislation, and Registration; Dr. Swearingen, on that of International and Public Hygiene; Dr. Hadra, on that of Gynecology; Dr. Ghent, on Collective Investigation, Nomenclature and Vital Statistics; Dr. J. F. Y. Paine, on that of Practical and Experimental Therapeutics, and Dr. John H. Pope, on Diseases of Women and Children.

The sub-committee will yet appoint for each State a finance Committee, to serve as a whole under a general chairman. The appointments from each State will consist of a chairman and as many members as there are congressional districts in that State.

Upon the whole, I think excellent work was done, and I heartily congratulate you upon the successful issue of the cause in which you were amongst the first to engage, and for which you made such a gallant stand, at New Orleans and in your Journal,—*the redistribution of the appointments upon a broader, wider and more fair and liberal basis*, and the overthrow of the *new code men.*

The committee will not meet again until May—at St. Louis—one day preceding the annual meeting of the American Medical Association. Yours Truly,

J. W. McLaughlin.

[We learn from Dr. McLaughlin, since his return to Austin, that he has appointed Dr. Jas. D. Osborn, of Cleburne, chairman of the Texas Finance Committee, with authority to select his members.—Ed.]

